# Head of household education level as a factor influencing whether delivery takes place in the presence of a skilled birth attendant in Busia, Uganda: a cross-sectional household study

**DOI:** 10.1186/1471-2393-13-48

**Published:** 2013-02-21

**Authors:** Frédérique Vallières, Alexandria Hansen, Eilish McAuliffe, Emma Louise Cassidy, Paul Owora, Sam Kappler, Evelyn Gathuru

**Affiliations:** 1 Centre for Global Health, Trinity College Dublin, 7-9 Leinster Street South, Dublin 2, Ireland; 2 World Vision Uganda, World Vision Uganda, Kisozi Complex, Kampala, Uganda; 3 World Vision Ireland, The Mews, Rathmines Park, Dublin 6, Ireland

**Keywords:** Skilled birth attendant, Health-centre delivery, Head of household, Education

## Abstract

**Background:**

Assistance during delivery by a skilled attendant is recommended as a means to reduce child and maternal mortality. Globally, higher levels of maternal education have been associated with better health behaviours at delivery. However, given that heads of households tend to be the decision makers regarding accessing healthcare, some educated mothers may find themselves prevented from accessing healthcare at the point of delivery.

**Methods:**

We examined the association between head of household education level and health seeking behaviours at delivery across a sample of 392 households. Chi-squared analysis and odds ratios were calculated to measure the strength of the relationship between no, some primary, or some secondary or higher education attained by the head of household and the presence or absence of a skilled birth attendant at that child’s birth, and whether the birth took place at a health facility.

**Results:**

Heads of household (n = 392) were predominantly male (93.4% [(90.9%, 95.8%), a = 0.05]). We found a significant difference in skilled birth attendance between heads of households with some primary education and heads of household with some secondary education or higher (*χ*^2^ (1) = 6.231, *p* <0.05) whereby those with secondary or higher education were significantly more likely to seek a skilled birth attendant (OR = 1.5,[1.1,2.1]). The difference in health centre delivery between heads of household with a primary education and heads of household with a secondary or higher education was also significant (*χ*^2^ (1) = 7.519, *p* <0.05). Those with secondary or higher education were significantly more likely to deliver in a health facility (OR = 1.6,[1.2,2.1]).

**Conclusions:**

The results of our analysis, which identified the vast majority of heads of households as men, suggests that education, or rather limited or a lack of education for the head of household, may be a barrier to women’s use of health care in Uganda and therefore reinforces the need to increase educational access among male heads of households. Improving the rates of health centre deliveries and utilization of services provided by skilled health workers might lie, in part, in increasing overall education levels of heads of households, specifically the education of male heads of households.

## Background

Deliveries attended by skilled providers such as nurses, doctors, or midwives, in hygienic conditions, can significantly reduce the risk of complications known to cause death or serious illness in both mothers and their children [1-8]. Increasing the number of women who seek a skilled birth attendant (SBA) remains one of the primary means advocated by the World Health Organization (WHO) to reduce maternal and neonatal mortality [9]. The last two decades have seen an increase in the rate of births assisted by SBAs worldwide, except in sub-Saharan Africa, where barriers still prevent women from accessing SBAs [10]. Such barriers include: lack of transportation, distance from the health centre, cost of travel and health services, perceived low-quality of care in facilities, unavailability of emergency obstetric care at health centres, and cultural barriers including women’s inability to travel alone and their adherence to traditional practices [2,11-15].

Uganda continues to rank among the countries with the highest maternal and child mortality. With an estimated Maternal Mortality Ratio (MMR) of 438/100,000 and an under five mortality rate of 90 per 1,000 live births, Uganda is not expected to meet Millennium Development Goals four or five [16,17]. The greatest risk of child death occurs around the time of birth and neonatal deaths in Uganda are estimated to account for nearly 53% of all infant deaths [17,18]. Despite important progress, the country’s most recent demographic health survey revealed that only 53% of births in rural areas were attended by skilled health personnel [17]. In Uganda’s Eastern region, 39.6% of births were accompanied by an unskilled assistant such as a traditional birth attendant (TBA), a relative or a friend, and 7.7% of births received no assistance at all [17].

Heads of households, who in most contexts tend to be predominantly male [19], often act as key decision makers in accessing skilled healthcare at the time of delivery and recent research suggests that husband-only decision making is negatively associated with skilled delivery care [20]. Specifically, men often provide the financial means, transport, and sometimes the permission for women to attend a health centre [12,15]. In Eastern Uganda, 58% of women delivering in a health facility were accompanied by their husband or partner [21]. Though it is widely agreed that men should be more involved in the continuum of care provided to pregnant women, research on how to best incorporate them in Uganda is lacking [22-25].

It is well established that maternal education is an important factor in improving child health outcomes, along with increasing overall household access to skilled healthcare. In Uganda, the neonatal mortality rate of infants whose mother had a primary education was found to be 10% lower than that of infants whose mother had no education [21]. Likewise, a study conducted in Entebbe, Uganda found that less educated mothers were more likely to give birth in the presence of an unskilled assistant or worse, with no assistance at all [11]. Globally, higher levels of maternal education have been associated with, amongst other things, increased knowledge and acceptability of vaccinations, better child immunization status, more consistent attendance of antenatal care visits, greater institutional deliveries, and reduced risk of neo-natal and post-neonatal deaths [11,26-29]. However, given that heads of households tend to be the decision makers regarding accessing healthcare, some educated young women and mothers may find themselves prevented from accessing healthcare. Previous studies have focused on the importance of maternal education and the important role heads of households (HOHs) play in accessing health care at delivery in Uganda.

## Methods

This paper first examines whether the education of the head of household (predominantly male and the key decision maker in Ugandan households) is associated with health seeking behaviours at time of delivery in Busia, Uganda. Where an association was found, we also further explore the strength of that association.

The secondary data obtained for this paper were collected as part of a baseline assessment of maternal and child health in the sub-counties of Busitema, Sikuda, Lunyo, and Busiime, located in Uganda’s Busia District, during June and July of 2011. World Vision Ireland and World Vision Uganda conducted the baseline in preparation for the implementation of a community-based maternal and child health programme using community health workers to target changes in household health behaviour. Bordering Kenya to the east and Tanzania to the south and formally known as Tororo District, Busia District has an estimated population of approximately 287,800 inhabitants [30].

### Sampling

The baseline exercise employed a cross-sectional household survey, which was conducted across a sample of 400 households located in four sub-counties of Busia. A two-stage probability sampling method was used to obtain a sample of the population in each parameter. Village lists were obtained for the sub-counties of Busitema, Sikuda, Lunyo, and Busiime. The probability of a village being selected was set as proportional to the number of households within that village. All households therefore had an equal chance of being selected regardless of whether they contained the target population or not.

In the second stage of sampling, village leaders led field teams to the village centre where a pen was spun to determine the field team’s walking direction. A random number generation table was subsequently used to decide which household was to be visited first. A total of 407 households, from 125 out of the possible 136 villages, were ultimately visited in the sample, 400 of which completed the questionnaire. Sample size was calculated assuming a confidence level of 95% (a = 0.05).

### Survey tool

The survey tool was adapted from the Ministry of Health’s (MOH) own village health team (VHT)/ICCM Register 2010 [31] and developed in consultation with the district health management team in Busia (Additional file 1). Though the questionnaire was printed in English, training was conducted in a mixture of English and Luganda. VHTs were permitted to conduct the interview in whichever language they felt best suited the household.

The household was defined in terms of any people who were co-resident and shared common cooking arrangements, and were able to recognise one person as the head of household [32]. Participants in each household were asked to identify the HOH, and that individual’s most recently completed education level. Participants were then asked to identify all children under the age of 5 within that household and the child’s relation to the HOH. For each child, subsequent questions determined the location of their birth (at a health centre or elsewhere), as well as who was present at the time of birth: a skilled provider, unskilled provider such as a TBA, both, or neither. Aligned with Ugandan MOH policy, a skilled provider was defined as a “doctor, nurse, midwife, medical assistant, or clinical officer” [21].

### Exclusion and inclusion criteria

To be considered for secondary analysis a household had to contain at least one child under the age of 60 months. Interviews were primarily conducted with the child’s primary caregiver. A primary caregiver was defined as the person who was, “primarily responsible for the health, safety and comfort of that child”. A total of 392 out of 400 de-identified households were included in the analysis.

### Ethical considerations

Informed written consent was obtained from all participants. If the participant was illiterate, signatures were obtained in the form of a fingerprint using an inkpad. Permission for the Centre for Global Health, Trinity College Dublin to use the de-identified baseline data for secondary analysis was obtained from both World Vision Ireland and World Vision Uganda and ethical approval was obtained from the Health Policy and Management/Centre for Global Health Research Ethics committee, Trinity College Dublin.

### Data analysis

Quantitative analysis was conducted using PASW Statistics 18 (Release Version 18.0.0) and SPSS Statistics 17 (Release Version 17.0.0). Delivery practices were statistically analyzed according to relevant demographic variables. Respondent’s level of education was recorded as the highest grade or year completed by that individual. For analysis, these were categorised as follows: no form of education; attained any level of primary education; or, attained any form of secondary or higher education. Both education levels and delivery practices were compared across age and gender to ensure comparability and to identify any possible confounders or effect modifiers. Descriptive tests and Chi-Square/Correlation analysis were used to demonstrate the effects of the independent variables on the probability of choosing a health centre facility for delivery, rather than remaining at home or outside a clinical setting for delivery. The effects of the independent variable on the probability of choosing assistance from a trained, skilled birth attendant versus opting for a traditional home delivery without trained assistance were also presented.

Pearson Chi-Square tests were conducted to measure the significance of the relationship between the education level attained by the HOH and the presence of an SBA or TBA (or other unskilled birth attendant) at that child’s birth, as well as whether the birth took place at a health facility. Additional Chi-Square tests of independence were individually executed on each possible combination of the groups. Where a statistically significant relationship was found, odds ratios were calculated with the lower education level as reference. Analysis was first conducted for children who were labeled as biologically related to the HOH. Biologically related children included only sons and daughters of the HOH. This same analysis was subsequently conducted for all children who were either biologically linked or relatively linked to the HOH. Relative children included nieces, nephews, stepchildren, and grandchildren. All tests were conducted for 95% confidence with α = 0.05.

## Results

### Characteristics of sample

HOHs in Busia (n = 392) were predominantly male (93.4% [(90.9%, 95.8%)]), with 71.2% of male HOHs aged 18 to 39 years ([66.6%, 75.9%]). Of the HOHs who responded to the education level question (n = 373), 5.6% had no form of education ([3.3%, 8.0%]), 58.7% had achieved some level of primary education [53.7%, 63.7%]), and 35.7% had completed at least some secondary level education or higher [30.8%, 40.5%]). Gender was related significantly to HOH education level (*χ*^2^ (2) = 59.751, p < 0.05), as was age (*χ*^2^ (2) = 6.761, p < 0.05), however neither of these factors was associated with attendants at delivery (SBA v. TBA) or place of delivery (health centre v. other). Adjustment via multinomial logistic regression was considered for age and gender of the HOH as both were associated with education level attained, however it had no effect on the outcome as neither were associated with delivery practices and indeed reduced the reliability of the model. The groups were therefore comparable with no need for adjustment by age or by gender.

Overall, 34.1% of the children under the age of five in Busia (n = 781) were delivered in the presence of a skilled provider ([30.7%, 37.4%]). In comparison, 57.4% of children under-five’s births (n = 779) were assisted by an unskilled provider (i.e. TBA) ([53.9%, 60.9%]), and 9.1% of children were delivered in the absence of any type of birth attendant ([7.1%, 11.1%], n = 779). Moreover, 37.9% of children (n = 781) were born in a health care facility ([34.5%, 41.3%]), while 62.1% were born elsewhere ([58.7%, 65.5%]).

### Relationship between HOH education level and assistance at birth of children biologically related to HOH

**Figure 1:**
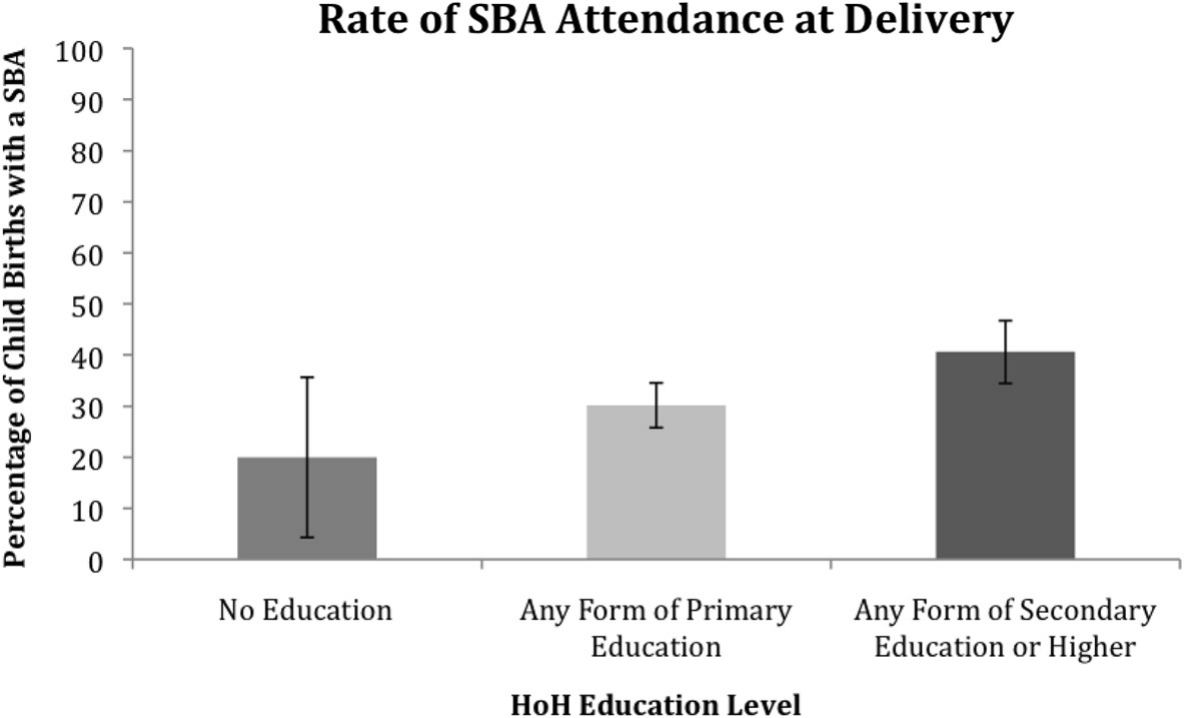
The utilisation of a skilled birth attendant during the birth of a child biologically related to the HoH according to the education level of that HoH.

For biologically related children, differences were observed in the utilisation of an SBA between HOHs with no education, those with primary education, and those with a secondary or higher education, see Figure 1. The Chi-Square test of independence revealed that the difference was statistically significant (*χ*^2^ (2) = 8.646, *p* <0.05). We found a significant difference in SBA attendance between HOHs with primary education and HOHs with secondary or higher education (*χ*^2^ (1) = 6.231, *p* <0.05) whereby those with secondary or higher education were significantly more likely to seek an SBA (OR = 1.5, [1.1,2.1]). The higher the education level the more likely the child’s birth had been attended by an SBA. The difference in SBA attendance between HOHs with no education and HOHs with primary education was not statistically significant.

**Figure 2:**
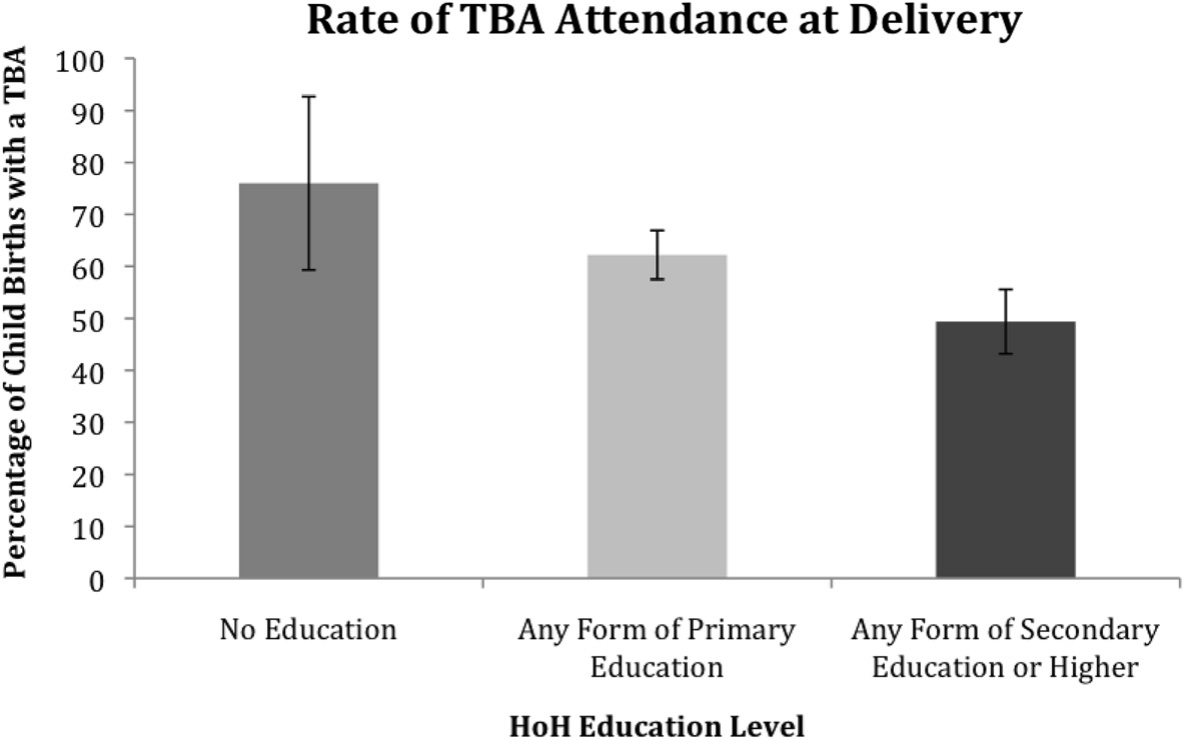
The utilisation of a traditional/unskilled birth attendant during the birth of a child biologically related to the HoH according to the education level of that HoH.

Differences were also observed in utilization of unskilled birth attendants, such as TBAs, between HOHs with no education, with primary education, and those with secondary or higher education, see Figure 2. The Chi-Square test of independence revealed that the difference was statistically significant (*χ*^2^ (2) = 12.296, *p* <0.05). The difference in unskilled attendance between HOHs with no education and HOHs with secondary or higher education was statistically significant (*χ*^2^ (1) = 4.768, *p* <0.05) whereby those with secondary or higher education were significantly less likely to seek an unskilled attendant (OR = 0.3, [0.1,0.8]); the difference in unskilled attendance between HOHs with primary education and HOHs with secondary or higher education was statistically significant (*χ*^2^ (1) = 8.646, *p* <0.05) whereby those with secondary or higher education were significantly less likely to seek an unskilled attendant (OR = 0.6, [0.5,0.8]); the difference in unskilled attendance between HOHs with no education and HOHs with primary education was not statistically significant.

**Figure 3:**
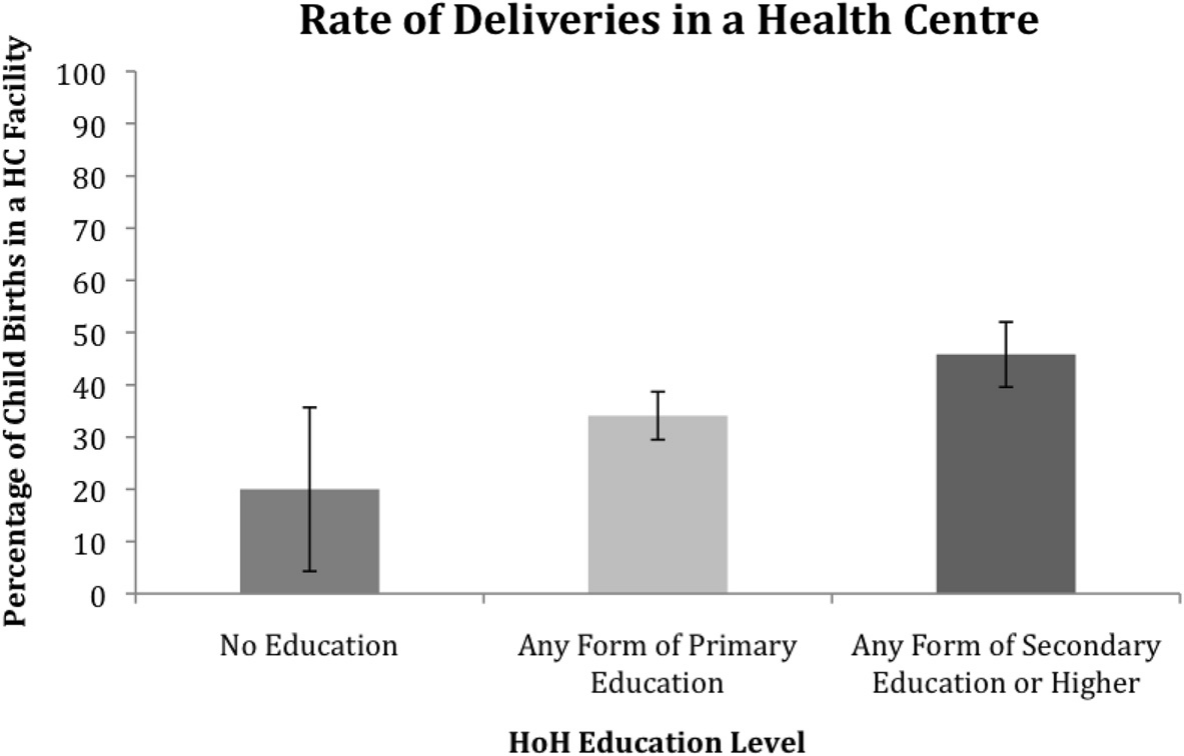
Births in a health care facility for children biologically related to the HoH according to the education level of that HoH.

Observed differences in whether the delivery took place at a healthcare facility for HOHs with no education, primary education, and those with secondary or higher education are depicted in Figure 3. The Chi-Square test of independence revealed that the difference was statistically significant (*χ*^2^ (2) = 11.256, *p* <0.05). The difference in health centre delivery between HOHs with no education and HOHs with secondary of higher education was statistically significant (*χ*^2^ (1) = 4.590, *p* <0.05) whereby those with secondary or higher education were significantly more likely to deliver in a health facility (OR = 3.2, [1.2,8.9]); the difference in health centre delivery between HOHs with primary education and HOHs with secondary or higher education was statistically significant (*χ*^2^ (1) = 7.519, *p* <0.05) whereby those with secondary or higher education were significantly more likely to deliver in a health facility (OR = 1.6, [1.2,2.1]); and the difference in health centre delivery between HOHs with no education and HOHs with primary education was not statistically significant.

### Relationship between HOH education level and assistance at birth of children (biological and relative) under-five in household

For all children, including biological and relative children, similar differences were observed in SBA attendance unskilled attendance and health centre delivery between HOHs differentiated by education levels. The Chi-Square test of independence revealed that differences in SBA attendance according to education level were statistically significant (*χ*^2^ (2) = 8.70, *p* <0.05); differences in unskilled attendance were statistically significant (*χ*^2^ (2) = 13.223, *p* <0.05); and delivery rates at a health centre were statistically significant (*χ*^2^ (2) = 14.062, *p* <0.05).

The difference in SBA attendance between HOHs with no education and HOHs with secondary or higher education was not statistically significant; the difference in SBA attendance between HOHs with primary education and HOHs with secondary or higher education was statistically significant (*χ*^2^ (1) = 7.908, *p* <0.05) whereby those with secondary or higher education were significantly more likely to seek a skilled attendant (OR = 1.6, [1.2,2.2]); and the difference in SBA attendance between HOHs with no education and HOHs with primary education was not statistically significant.

Neither the difference in presence of an unskilled birth attendant between HOHs with no education and HOHs with secondary or higher education nor the difference in presence of an unskilled birth attendant between HOHs with no education and those with primary education were found to be significant. However, the difference in the presence of an unskilled birth attendant between HOHs with primary education and HOHs with secondary or higher education was significant (*χ*^2^ (1) = 10.442, *p* <0.05) whereby those with secondary or higher education were significantly less likely to seek assistance from unskilled personnel (OR = 0.6, [0.5,0.8]). The difference in health centre delivery between HOHs with no education and HOHs with secondary or higher education was statistically significant (*χ*^2^ (1) = 5.727, *p* <0.05) – those with secondary or higher education were significantly more likely to deliver in a health facility (OR = 2.6, [1.2,5.6]); the difference in health centre delivery between HOHs with primary education and HOHs with secondary or higher education was statistically significant (*χ*^2^ (1) = 10.425, *p* <0.05) whereby those with secondary or higher education were significantly more likely to deliver in a health facility (OR = 1.7, [1.3,2.3]); and the difference in health centre delivery between HOHs with no education and HOHs with primary education was not statistically significant.

Overall, our analysis shows that there is an association as well as a significant effect between education level of the HOH and health-seeking behaviour at the time of delivery.

## Discussion

Children born in households in which the HOH had an education level beyond that of primary education were more likely to be born in a health facility and in the presence of a skilled provider. Specifically, a child whose HOH had some form of secondary or higher education sought health services for delivery at significantly higher rates than either those born in households in which the HOH had only a primary education or those born in households in which the HOH had no education. Our results further indicate that simply attaining a primary education did not have a significant impact on the rates of SBA attendance and health centre deliveries. Regardless of the child’s relationship to the HOH, whether biological or relative, the observed association relating to education remained significant.

The association between education and health behaviours reinforces the significant role that education plays in improving utilisation of maternal health services. Though the relationship between a mother’s education level and delivery practices is well established throughout the existing literature, this study suggests that the education level of the decision maker (i.e. head of household) is also a significant factor in women’s utilisation of health services. Given that one of the primary international recommendations for reducing maternal and child mortality is increasing access to skilled health providers, prioritization in Uganda needs to be given to increasing health centre attendance through increasing education [33]. Specifically, emphasis is needed on promoting education for those who are thought to play a critical role in the health behaviour decision-making process. In Uganda, where the majority of households are headed by men, this means not just focusing on young girls and women’s education, but also educating young men as future HOHs, and thus key decision makers on access to maternal health care.

A study conducted in Uganda’s southern Rakai district, found that women who felt that attending the health centre was their decision alone were reportedly more likely to deliver in health units compared to those who said that attending health centres depends on their husbands [12]. Current strategies in Uganda tend to primarily focus on women, even though it is often men who provide the financial support and play an influential role in the health-seeking process [15].

The results of our study, which identified the vast majority of HOHs as men, suggests that education, or rather limited or a lack of education, may be a barrier to the use of health care in Uganda and therefore reinforces the need to increase educational access among male HOHs. It is unclear however, whether higher male education levels mediate better health seeking behaviours at the time of delivery through providing women with information as well as more control over their decision on where to access health care, or through greater male dominance. Education levels for male HOHs may increase access to skilled deliveries particularly where fees might be incurred because male HOHs have greater control over household finances and can accordingly manage and regulate the seeking of health services [34]. Though level of income was not quantified in the survey, greater levels of education could account for greater household income, and those with higher incomes are more likely to seek access to healthcare services [35]. The availability of TBAs, that they are often from the same locality, and decreased financial costs associated a TBA assisted delivery, must also be considered as factors influencing the preference of TBAs at the point of delivery in Uganda [36]. Secondary data obtained being largely quantitative, we were unable to account for the influence of cultural and religious beliefs on delivery services in our analysis, which acts as a limitation to our study. Similarly, the secondary data obtained only recorded the relationship between children under-five to the HOH, implying that we were unable to link delivery practices to educational levels of mothers.

While maternal education has been identified as a factor influencing health centre births and overall utilisation of maternal health services, strengthening the education of male HOHs may be equally, if not more, important in improving child and maternal health in Uganda. More explorative studies describing the pathways and examining the processes through which male HOH education influences whether or not a woman gives birth in the presence of a skilled birth attendant are necessary. As with previous studies, which found an association between higher rates of male attendance at antenatal clinics and higher levels of education, and with reviews showing that a lack of paternal involvement negatively affected pregnancy outcomes, there is a need to increase male participation in both education and maternal and child health promotion programmes [23,37]. As millions of women within South Asia and sub-Saharan Africa continue to give birth without an SBA over the next few years [38], a more educated, more knowledgeable HOH is likely to be a critical component in overcoming existing barriers and further increasing the percentage of births attended by skilled birth attendants.

## Conclusions

Access to high quality maternal and child health services are essential to lowering maternal and child mortality. However, more attention should be paid to the household factors that may mediate access to such health services. Strategies to increase education levels amongst men, combined with providing men with access to knowledge about maternal and child health services may contribute to increasing skilled attendance at delivery, and to the improvement of maternal health outcomes overall.

## Abbreviations

SBA: Skilled birth attendant; TBA: Traditional birth attendant; HOH: Head of household; MMR: Maternal mortality ratio; VHT: Village health team.

## Competing interests

The authors declare that they have no competing interests.

## Authors’ contributions

FV & AH initiated the concept for the use of the data, came up with the analysis plan, and co-wrote the manuscript. AH conducted the data analysis and FV contributed towards the design. EM reviewed and provided substantial inputs into the manuscript review. ELC provided thorough statistical review and further data analysis. PO, EG & SK played key roles in the tool development, implementation, and data collection phases. All authors read, provided substantial input, and approved the final manuscript. FV is the final guarantor of the paper. All authors read and approved the final manuscript.

## Pre-publication history

The pre-publication history for this paper can be accessed here:

http://www.biomedcentral.com/1471-2393/13/48/prepub

## Supplementary Material

Additional file 1 Household register. Click here for file
