# Supplementary material for: Head of household education level as a factor influencing whether delivery takes place in the presence of a skilled birth attendant in Busia, Uganda: a cross-sectional household study
Source: BMC Pregnancy Childbirth. 2013 Feb 21;13:48. doi: 10.1186/1471-2393-13-48 (PMC3623753; doi:10.1186/1471-2393-13-48)
Supplement: Additional file 1 — Household register. [file 1471-2393-13-48-S1.pdf]

HOUSEHOLD REGISTER

(i) Survey Number___________________	(iii) Village _______________ Circle: LUNYO or BUSITEMA	(v) Name of Person completing interview ________________________
(ii) Name of VHT member: _____________	(iv) Reporting Period (Date) _______________	(vi) Head of Household _____________________

(vii) RC Present____

(viii) Referral (give reason) _________________

(ix) Number of People in the Household__________

Supervisor__________________

Adults & Children Above 5

GENERAL INFO: Adults										PREGNANCY												MALARIA		NUTRITION						DANGER SIGNS				
Q1	Q2		Q3	Q4	Q5	Q6	Q7	Q8	Q9	Q10	Q11	Q12				Q13	Q14	Q15	Q16	Q17	Q18	Q19	Q20	Q21	Q22					Q23	Q24	Q25	Q26	Q27
Household Members (above 5 Years)	Sex		Age (yrs)	Relationship to HHH	Death(past 1 yr)	Disability?	Source of Income	Level of Education	Child in School (Under 18)	Pregnant?	Delivery Month	ANC Visits				TT given?	Dewormed?	Access to PMTCT	Received HIV info during ANC	Tested as part of ANC?	Received Results	Slept under ITN last night	Does net have holes?	# Meals Yesterday	Starches	Meat and Dairy	Legumes	Greens	Fruit	# Pregnancy Danger signs	# Neonatal Danger Signs	# Methods of MTCT	# Post Partum Danger signs	# Danger Sign Pneumonia
	M	F										1	2	3	4																			
																																		RESPONDENT ONLY				

Children 5 Years and Below

General Information							Pregnancy Care								PNC			Immunisation & Supplements																				Malaria		Nutrition							
Q28	Q29		Q30	Q31	Q32	Q33	Q34	Q35	Q36	Q37	Q38	Q39	Q40	Q41	Q42	Q43	Q44	Q45	Q46a	46b	46c	46d	46e	46f	46g	46h	46i	46j	46k	46l	46m	46n	46o	Q47		Q48		Q49	Q50	Q51	Q52	Q53					Q54
Household Members (5 Years and below)	Sex		Age (months)	Relationship to HHH	Death (past 1 yr)	Disability?	Delivered with SBA?	Delivered with TBA?	Delivered in HC	Birth Certificate?	Received HIV info during ANC	Tested as part of ANC?	Received Results	Received Counselling	6 Hours	6 Days	6 Weeks	Has Card?	BCG	OPV 0	OPV 1	DPT-Heb B+Hib 1	PCV 1	Rota 1	OPV2	DPT-Heb B+Hib 2	PCV 2	Rota 2	OPV 3	DPT-Heb B+Hib 3	PCV 3	Rota 3	Measles	Worm		Vit A		ITN	Net with holes	Exclusive Breastfeeding	# Meals Yesterday	Starches	Legumes	Meat	Greens	Fruit	MUAC (G,Y,R)
	M	F																																1st	2nd	1st	2nd										
																																																Birth		6 Weeks				10 Weeks				14 Weeks				9 Months														

Survey Number__________________

ICCM SICK PATIENT REGISTER FOR CHILDREN 5 YEARS AND BELOW

GENERAL INFO				PROBLEM (past 2 weeks)					TREATMENT				
Q55	Q56		Q57	Q58	Q59	Q60	Q61	Q62	Q63	Q64	Q65	Q66	Q67
Patient Name	Sex		Age in months	Diarrhoea	Fast Breathing	Cough	Fever	Treated at HC within 24 hrs	ORS	Breastfeeding	Home Solution	Tablets	Traditional Remedy
	M	F											

HOUSEHOLD

WATER AND SANITATION								
Q68	Q69	Q70	Q71	Q72				
Time Spent Fetching Water (MINS)	# Litres per Day	Latrine is Clean	Water Treatment	*Before Eating*	*After Changing Baby*	*After Latrine*	*Before Food Prep*	*Before Breastfeeding*
				5 CRITICAL TIMES				

FIELD NOTES

HOUSEHOLD: Water, Food and Sanitation

Find out if the household has unlimited access to the following methods to prevent sickness. Tick the boxes for the things the family has:

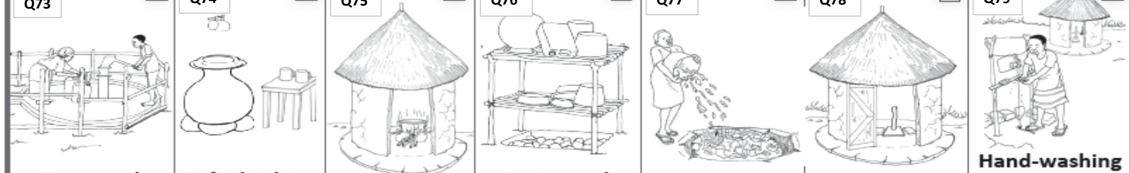

Q73 ☐	Q74 ☐	Q75 ☐	Q76 ☐	Q77 ☐	Q78 ☐	Q79 ☐
**Protected water source**	**Safe drinking water**	**Kitchen**	**Drying rack for dishes**	**Rubbish pit**	**Latrine**	**Hand-washing area with soap near latrine**

Follow up on anything the family is missing.
